# A comparative study of three methods for detecting association of quantitative traits in samples of related subjects

**DOI:** 10.1186/1753-6561-3-s7-s122

**Published:** 2009-12-15

**Authors:** Aude Saint Pierre, Zulma Vitezica, Maria Martinez

**Affiliations:** 1 INSERM, U.563, University Paul-Sabatier, CPTP, Toulouse F-31300, France

## Abstract

We used Genetic Analysis Workshop 16 Problem 3 Framingham Heart Study simulated data set to compare methods for association analysis of quantitative traits in related individuals. More specifically, we investigated type I error and relative power of three approaches: the measured genotype, the quantitative transmission-disequilibrium test (QTDT), and the quantitative trait linkage-disequilibrium (QTLD) tests. We studied high-density lipoprotein and triglyceride (TG) lipid variables, as measured at Visit 1. Knowing the answers, we selected three true major genes for high-density lipoprotein and/or TG. Empirical distributions of the three association models were derived from the first 100 replicates. In these data, all three models were similar in error rates. Across the three association models, the power was the lowest for the functional SNP with smallest size effects (i.e., α2), and for the less heritable trait (i.e., TG). Our results showed that measured genotype outperformed the two orthogonal-based association models (QTLD, QTDT), even after accounting for population stratification. QTDT had the lowest power rates. This is consistent with the amount of marker and trait data used by each association model. While the effective sample sizes varied little across our tested variants, we observed some large power drops and marked differences in performances of the models. We found that the performances contrasted the most for the tightly linked, but not associated, functional variants.

## Background

For pedigree-based association analysis, several methods have been developed that utilize information about transmission of alleles, such as the orthogonal test for within-family variation (quantitative transmission-disequilibrium test, or QTDT) [1,2]. The quantitative trait linkage-disequilibrium test (QTLD) is a modification of the QTDT method that assigns the founder genotypes to the within-family component rather than to the between-family component [3]. The measured genotype (MG) model is a simple fixed-effects regression for which non-independence in the data is accounted for by polygenic effects [4,5]. All three approaches, QTDT, QTLD, and MG, can be applied to the association analysis of quantitative traits in extended pedigrees. They differ in the amount and type of marker information used for testing association. The MG model uses all individuals with available phenotype and genotype data. The family-based models use a subset of this sample. The effective sample size of QTDT is further reduced because founders and spouses are not use to estimate the within-component effect. Thus, QTDT may lack of power compared with QTLD and/or MG but, on the other hand, both MG and QTLD tests may be affected by allelic association due to population stratification. The relative merit of these approaches has been investigated in a few instances [3,6]. Here, we extend these studies to explore type I error and relative power of QTDT, QTLD, and MG tests in a large pedigree-based sample, i.e., Genetic Analysis Workshop 16 Problem 3 Framingham Heart Study (FHS) simulated data set. Our investigation was performed with knowledge of the answers.

## Methods

### Choice of the quantitative traits studied for association analysis

We studied the two simulated quantitative traits, high-density lipoprotein (HDL) and triglyceride (TG), measured at Visit 1 in FHS simulated data set. All our analyses were conducted using the first 100 replicates. Within each replicate, we adjusted trait values for sex and age using a linear regression. We used the residual values of HDL and TG as the phenotypes of interest for association testing. We then assessed the distributions of each trait using the 100 replicates. We found that HDL, but not TG (kurtosis = 16.21, skewness = 2.49), values were normally distributed. The fit to the normal distribution was obtained using a rank-based transformation of TG values (TG_Rob): kurtosis and skewness were equal to -0.02 and 0.003, respectively.

### SNP data preprocessing

Genotype data were obtained from the Affymetrix GeneChip Human Mapping 500 k Array. Individual genotype data were filtered based on BRLMM (Bayesian robust linear model with Mahalanobis distance) confidence scores: we used the standard cutoff of 0.5 for call/no-call. Quality control analyses led to 1) exclusion of SNPs with less than 95% call rates, with unknown map position, or with low minor allele frequency (<1%); 2) zeroing out all genotypes at any DNA sample with <95% call rate; 3) exclusion of SNPs not fitting the Hardy-Weinberg equilibrium (*p*-value ≤ 10^-6^) hypothesis; (4) zeroing out genotypes of all individuals in a family at any SNP that showed mendelian errors.

### Pedigree sample data

From the total FHS sample of 940 pedigrees, we selected 704 pedigrees having at least two non-founders individuals with available phenotype and genotype data.

### Choice of the SNPs tested for association

In brief, the simulation models for HDL and TG included the effects of major genes (five for HDL and three for TG, each explaining 0.1-0.3% of the total variance), and polygenic effects (58% for HDL and 38% for TG). Here, we limited our study to three (*LPL*, *CYP2B7P1*, and *CYP2B6*) of the HDL major genes. TG variability was also explained by two (*LPL *and *CYP2B6*) of these genes. Table 1 lists the main characteristics of all studied SNPs; within each gene the functional SNP is denoted with its symbol name, *h*^2^*g *is the rate of the trait variance explained by each functional SNP, and *D*' is the standardized Lewontin's disequilibrium coefficient between the functional variant and each SNP being tightly linked to it. The total number of subjects with available phenotype and genotype data ranged from 5,826 to 5,995. Note that two functional SNPs (α2 and δ1) are tightly (~120 kb) linked but not associated (*D*' = 0.003). For each gene, we used the functional SNP and two "non-associated" SNPs, selected from the set of SNPs tightly linked (<100 kb) and not associated (*D*' < 0.10) to the functional SNP. Finally, we also investigated association tests using SNPs not linked to any of the functional variants. The 'false' gene was randomly drawn on chromosome 7 (position: 24,734,008 bp).

**Table 1:** Characteristics of the SNPs selected to test association to HDL and TG

							*h* ^2^ *g*	
							
Chr	Gene	Position (bp)	SNP	MAF (%)	Symbol	D' (functional variant)^a^	HDL	TG
7	None	24,734,008	rs2521760	12.7	-	-	-	-
8		19,794,163	rs17091651	10.0	-	0.04 (α4)	-	-
	*LPL*							
		19,868,351	rs3200218	21.7	α4		0.3%	0.4%
		19,943,326	rs4244457	32.9	-	0.04 (α4)	-	-
								
19		46,010,146	rs11083567	18.2	-	0.07 (α2) - 0.03 (δ1)	-	-
	*CYP2B7P1*	46,089,501	rs8103444	24.4	α2	0.003 (δ1)	0.2%	
	*CYP2B6*	46,210,613	rs8192719	24.9	δ1	0.003 (α2)	0.3%	0.3%
		46,335,684	rs1631931	13.5	-	0.01 (α2) - 0.03 (δ1)	-	-

^a^Pairwise linkage disequilibrium coefficient (D'/Dmax) between the functional variant (symbol) and the SNPs in its vicinity (<200 kb)

### Pedigree-based association tests

All association analyses were performed using the qtld command of SOLAR 4.0.7 [7]. The QTDT model decomposes marker effects into two orthogonal components: the between- (bb) and the within- (bw) family components [1]. The restricted model depends on bb only (bw is set to 0). Significance of association is assessed by computing the likelihood ratio of the restricted vs. unrestricted model. The QTLD model [3] includes the founder genotypes in the within-family component rather than in the between-family component (denoted as *b*'*w *and *b*'*b*). Restricted and unrestricted likelihoods of both the QTDT and QTLD models were maximized as a function of the polygenic component (*h*^2^). The MG model is a classical mixed model in which the marker is included as a covariate, and the correlations between relatives are accounted for by *h*^2 ^[5]. The regression coefficient of the marker (*b*) is the association parameter. The restricted MG model depends on *h*^2 ^only (*b *set to 0). The SNP was coded as the number of rare allele copies across all three methods. The effective sample sizes of these three association tests differ. MG model uses all subjects (founders, spouses, and relatives) with known phenotype and genotype status. From this sample, the two family-based association models discard data on the relatives not fulfilling either one of the two conditions: 1) their two parents are genotyped and at least one of them is heterozygote or 2) they have at least one sibling with a different genotype. The effective sample size of QTDT is further reduced because founders and spouses are not use to estimate the within-component effect.

Evidence for population stratification (denoted here as STRAT) is assessed through the likelihood ratio of the restricted (bw and bb are held equal) to the unrestricted (bw and bb are estimated freely) model. All three association tests, as well as the STRAT test, are assumed to follow a chi-square distribution with one degree of freedom.

We performed single-SNP association analyses. For each trait and each SNP, we computed the three association tests (and STRAT test) in each replicate, and derived the mean and standard deviation of each chi-square statistic over 100 replicates. We also derived mean and standard deviations of the association parameters (regression coefficients). Power and error rates were defined as the proportion of replicates with a chi-square value exceeding a given nominal threshold value. MG and QTLD analyses were also performed accounting for population stratification (denoted as MG_S and QTLD_S tests): MG and QTLD chi-square values were both set to zero in the replicates having a STRAT *p*-value ≤ 5%. The three association tests were evaluated under varying conditions regarding i) inclusion or exclusion of the dietary effect (covariate "diet" affects TG levels and is correlated among family members) and in the association model, ii) trait distribution, i.e., untransformed vs. transformed trait values. Indeed, these association models assume that trait values are normally distributed, and departures from normality can inflate their type I error or reduce their power.

## Results and discussion

Table 2 shows empirical estimates of the mean chi-square statistics and type I error rates, at a nominal *p*-value of 5%, of QTDT, QTLD, and MG tests when the studied SNP is not associated to the trait. The three association tests were roughly similar in empirical estimates, whether or not the studied SNP is linked to a major gene. In general, error rates were lower or close to the nominal values, except for QTDT with two SNPs. As expected, accounting for population stratification decreased the mean chi-square statistics of both QTLD and MG models. Interestingly, in these data, departure from normality did not yield inflated error rates, except with QTDT for TG and rs4244457. Error rates remained unchanged when diet was included into the model (results not shown).

**Table 2:** Mean χ^2 ^statistics (μ-χ^2^) and type 1 error rates (at a nominal *p*) of QTDT, QTLD, and MG association tests

			μ-χ^2^(SD)					*p *= 5%		
				
Trait	Gene	SNP	QTDT	QTLD	QTLD_S	MG	MG_S	QTDT	QTLD_S	MG_S
A. No association and no linkage										
HDL	none	rs2521760	0.48 (0.62)	0.82 (1.00)	0.82 (1.00)	0.73 (0.85)	0.72 (0.86)	0%	1%	0%
TG			0.86 (1.10)	1.00 (1.15)	0.97 (1.15)	0.64 (0.70)	0.60 (0.69)	3%	4%	1%
TG_Rob			0.99 (1.28)	0.92 (1.24)	0.76 (1.03)	0.48 (0.63)	0.44 (0.62)	4%	4%	0%
B. No association and linkage										
HDL	*LPL*	rs17091651	1.03 (1.12)	1.36 (1.51)	1.36 (1.51)	0.93 (1.01)	0.93 (1.01)	2%	7%	3%
		rs4244457	1.50 (1.79)	1.03 (1.36)	0.60 (0.92)	0.63 (0.79)	0.51 (0.75)	6%	1%	1%
	*CYP2B7P1/*	r11083567	0.85 (0.97)	0.48 (0.63)	0.38 (0.53)	0.40 (0.51)	0.37 (0.52)	2%	0%	0%
	*CYP2B6*	rs1631931	1.86 (2.02)	0.72 (1.09)	0.61 (0.85)	1.08 (1.24)	0.99 (1.19)	12%	1%	1%
TG	*LPL*	rs17091651	0.70 (0.88)	0.93 (1.31)	0.88 (1.32)	0.89 (1.10)	0.83 (1.12)	1%	4%	5%
		rs4244457	1.76 (1.86)	1.27 (1.46)	0.99 (1.32)	0.65 (1.13)	0.61 (1.12)	14%	5%	3%
	*CYP2B6*	rs11083567	0.75 (0.95)	1.11 (1.22)	1.03 (1.18)	0.55 (0.77)	0.53 (0.77)	1%	3%	2%
		rs1631931	0.93 (1.35)	1.19 (1.41)	1.10 (1.33)	0.62 (0.79)	0.61 (0.79)	6%	7%	1%
TG_Rob	*LPL*	rs17091651	0.75 (0.99)	0.89 (1.22)	0.77 (1.16)	0.80 (0.89)	0.74 (0.89)	2%	3%	1%
		rs4244457	1.52 (1.74)	0.75 (1.20)	0.66 (1.20)	0.52 (0.98)	0.49 (0.99)	9%	4%	3%
	*CYP2B6*	rs11083567	0.73 (1.03)	0.66 (1.15)	0.66 (1.16)	0.5 (0.69)	0.5 (0.69)	2%	1%	0%
		rs1631931	0.97 (1.29)	0.85 (1.04)	0.79 (0.98)	0.75 (0.93)	0.74 (0.93)	2%	2%	1%

Table 3 shows empirical estimates of the three association models when the studied SNP is the functional polymorphism. For QTLD and MG models, we chose to report the results obtained after accounting for population stratification. Across the three association models, the power was the lowest for the functional SNP with smallest size effects (i.e., α2), and for the less heritable trait (i.e., TG). For TG, mean chi-square estimates were slightly increased when diet was included into the model. For QTDT and MG_S models, estimates were also increased when the trait was normal (i.e., using TG_Rob), relative to when the trait was non-normal. Reverse trends were observed for QTLD_S. The direction of the association parameters was consistent across the three association models (results not shown). Overall, for a given trait and SNP, the mean chi-square statistic was always the highest with MG_S and the lowest with QTDT. The mean chi-square of QTDT was 1.6 to 6.2 times lower than that of MG_S. For QTLD_S the ratios ranged from 1.0 to 2.4. This is consistent with the amount of marker and trait information used by each association model. For MG, the effective sample sizes (Ne) ranged from 5854 (α4) to 5995 (α2 and δ1) subjects. For QTLD and QTDT, Ne values ranged from 2436 (α4) to 2839 (δ1), and from 1846 (α4) to 2240 (δ1), respectively. It is worth noting that across the three functional variants, the drops in Ne values relative to that of MG varied little: they were the lowest with δ1 (2.11 vs. 2.68 for QTLD vs. QTDT), and the highest with α4 (2.40 vs. 3.17 for QTLD vs. QTDT). In contrast, and for HDL, differences in the performances of the models were more marked with δ1 than with α4. Indeed, mean chi-square statistic of QTLD_S was 1.73 lower than that of MG_S with δ1, whereas both tests showed same performances with α4. Similarly, drops of the mean QTDT statistic, relative to MG_S, were much greater with α2 (6.25) or δ1 (2.37) than with α4 (1.56). It is worth noting that α4 and δ1 explained similar amount of HDL variability. Thus, these results suggest that the relative performance of the association models can not be simply related to differences in the effective sample sizes.

**Table 3:** Mean χ^2 ^statistics (μ-χ^2^) and power (at a nominal *p*) of QTDT, QTLD, and MG

		μ-χ^2 ^(SD)			*p *= 5%			*p *= 0.1%		
				
SNP symbol	Trait	QTDT	QTLD_S	MG_S	QTDT	QTLD_S	MG_S	QTDT	QTLD_S	MG_S
α4	HDL	17.88 (6.28)	27.18 (11.51)	27.88 (11.55)	100%	91%	91%	89%	91%	91%
	HDL_Diet	17.87 (6.28)	27.17 (11.51)	27.88 (11.54)	100%	92%	92%	91%	92%	92%
α2	HDL	1.38 (1.35)	3.56 (2.59)	8.62 (5.05)	7%	46%	83%	0%	1%	34%
	HDL_Diet	1.38 (1.35)	3.56 (2.59)	8.62 (5.05)	7%	46%	83%	0%	1%	34%
δ1	HDL	7.13 (3.80)	9.77 (4.48)	16.90 (6.00)	80%	95%	99%	15%	35%	92%
	HDL_Diet	7.13 (3.80)	9.76 (4.48)	16.89 (6.00)	80%	95%	99%	15%	35%	91%
										
α4	TG	2.21 (2.46)	5.88 (4.67)	9.92 (6.31)	16%	59%	83%	2%	11%	43%
	TG_Diet	2.21 (2.35)	6.01 (4.6)	10.19 (6.07)	22%	62%	86%	2%	14%	46%
	TG_Rob	3.35 (3.16)	4.67 (4.11)	12.67 (6.34)	33%	46%	94%	3%	10%	58%
δ1	TG	3.11 (2.87)	7.97 (5.29)	12.13 (6.16)	28%	78%	92%	2%	26%	52%
	TG_Diet	3.11 (2.87)	8.04 (5.42)	12.28 (6.22)	32%	77%	92%	3%	26%	54%
	TG_Rob	5.15 (3.58)	7.46 (4.64)	17.46 (7.04)	57%	75%	95%	11%	21%	86%

In conclusion, our results showed that MG outperformed the two orthogonal-based association models (QTLD, QTDT), even after accounting for population stratification. QTDT had the lowest power rates. Similar conclusions were reached by two previous simulation studies [3,6]. It is worth noting that our investigation was conducted in a relatively large pedigree-based sample (>5,850 subjects with known phenotype and genotype status; out of these ~10% are founders). Thus, although the major gene-specific effects were very modest (<0.4%), the three association models showed good power (>80%, at *p *= 5%) to detect direct association for HDL and two (α4 and δ1) of the three functional variants. At a lower tabulated threshold (*p *= 0.1%), the power remained good using the MG model only. For TG, good power was obtained with the MG model with one functional SNP (δ1) and using transformed TG values.

## List of abbreviations used

FHS: Framingham Heart Study; HDL: High-density lipoprotein; QTLD: Quantitative trait linkage disequilibrium; QTDT: Quantitative transmission-disequilibrium test; MG: Measured genotype; SNP: Single-nucleotide polymorphism; STRAT: Population stratification; TG: Triglyceride.

## Competing interests

The authors declare that they have no competing interests.

## Authors' contributions

ASP carried out the statistical genetic analyses and drafted the manuscript. ZV contributed in the statistical analysis and helped to draft the manuscript. MM conceived the study, coordinated it, and contributed to the draft the manuscript. All authors read and approved the final manuscript.
